# Oncogenic viruses and their role in oral cavity and oropharyngeal squamous cell carcinoma: a systematic review

**DOI:** 10.3389/fonc.2026.1806640

**Published:** 2026-07-21

**Authors:** Juan Manuel Quiroga-Garza, Maria Fernanda Herrera-Saldivar, Norma Carolina Hernandez-Bautista, Alondra Yamileth Alanis-Valdez, Aldo Sebastian Flores-Flores, Jose Manuel Vazquez-Guillen, Diana Resendez-Perez, Reyes S. Tamez-Guerra, Cristina Rodriguez-Padilla

**Affiliations:** 1 Universidad Autónoma de Nuevo León, Facultad de Ciencias Biológicas, Laboratorio de Inmunología y Virología, San Nicolás de los Garza, Nuevo León, Mexico; 2 Universidad Autónoma de Nuevo León, Facultad de Ciencias Biológicas, Departamento de Biología Celular y Genética, San Nicolás de los Garza, Nuevo León, Mexico

**Keywords:** head and neck squamous cell carcinomas, oncogenic viruses, oral squamous cell carcinoma, oropharyngeal squamous cell carcinoma, systematic review, tumor virus infections

## Abstract

Oral cavity (OC) and oropharyngeal (OP) squamous cell carcinoma (SCC) represent a major global health concern, accounting for most of head and neck malignancies. These tumors arise from the epithelial lining of the OC and oropharynx and are characterized by aggressive local invasion, lymph node metastasis, and high morbidity and mortality. In addition to known risk factors such as tobacco and alcohol consumption, several oncogenic viruses, including human papillomavirus (HPV), Epstein-Barr virus (EBV), human herpesviruses (HHV) 1 and 2, cytomegalovirus (CMV) and Merkel cell polyomavirus (MCPyV), have been implicated in the development, progression, or prognosis. Detection of viral genomes within tumor tissues suggests a complex interplay between viral oncogenes, host immune responses, and environmental factors in carcinogenesis. This systematic review synthesizes current evidence on the contribution of oncogenic viruses to the etiology of OC and OP SCC. Following the PRISMA 2020 guidelines, observational studies between January 2015 and December 2025 were identified through searches of PubMed, Scopus and Web of Science. Eligible studies were limited to human research and reporting viral detections. The results demonstrate a strong, consistent, and clinically relevant association between HPV infection and OP SCC, while evidence supporting a causal or prognostic role for non-HPV oncogenic viruses in OC and OP SCC remains limited and heterogeneous. Substantial methodological variability in viral detection approaches and epidemiological reporting was observed, underscoring the need for standardized assessment frameworks. Overall, these results support site-specific interpretation of viral oncogenesis and inform the development of targeted prevention, diagnostic stratification, and therapeutic strategies.

## 1 Introduction

Oral cavity (OC) and oropharyngeal (OP) squamous cell carcinomas (SCC) represent a major global health burden and rank among the most common malignancies worldwide. More than 90% of head and neck cancers arise from squamous epithelium. Despite sharing histopathological features, OC and OP SCC differ substantially in their etiological drivers, biological behavior, and clinical outcomes, underscoring the need for site-specific evaluation of carcinogenic mechanisms. In recent decades, oncogenic viruses have emerged as critical modifiers of disease incidence, prognosis, and therapeutic response in subsets of head and neck squamous cell carcinoma (HNSCC)( (1, 2).

Human papillomavirus (HPV)-positive OP SCC has been recognized as a distinct clinical and molecular entity, characterized by rising incidence, younger age at diagnosis, and significantly improved survival compared with HPV-negative disease (3). Large population-based studies consistently demonstrate that HPV positivity confers a favorable prognostic effect in OP SCC, independent of traditional risk factors such as tobacco and alcohol exposure (4–6). These observations have directly influenced contemporary staging systems, including the 8th edition of the Union for International Cancer Control and the American Joint Committee on Cancer (UICC/AJCC) classification, which incorporates HPV status as a key determinant of prognosis and stage grouping in OP SCC (7, 8).

### 1.2 Biological mechanisms of HPV-driven carcinogenesis

At the molecular level, HPV-driven carcinogenesis in OP SCC is characterized by complex virus-host interactions, involving alterations in cell-cycle regulation, tumor suppressor pathways, and transcriptional programs. Integrative genomic and transcriptomic studies have identified differential expression of oncogenic pathways, immune-related genes, and potential therapeutic targets in HPV-positive tumors, further supporting their biological divergence from HPV-negative counterparts (9). Although HPV16 remains the predominant genotype in OP SCC, increasing detection of other high-risk HPV types has been reported, with emerging evidence suggesting genotype-specific differences in age distribution, tumor characteristics, and potentially clinical outcomes (6, 10–12).

The oncogenic potential of high-risk HPV is primarily mediated by the viral proteins E6 and E7, which promote malignant transformation through functional inactivation of the p53 and retinoblastoma (pRb) tumor suppressor pathways (13). In addition, the E5 oncoprotein contributes to carcinogenesis by enhancing epidermal growth factor receptor (EGFR) signaling, promoting cellular proliferation, and facilitating immune evasion. The viral proteins E1 and E2 are essential for viral replication and genome maintenance; notably, disruption of E2 following viral integration into the host genome can result in deregulated E6 and E7 expression, thereby accelerating malignant progression (14). Consequently, persistent HPV infection leads to cell-cycle dysregulation, genomic instability, and uncontrolled cellular proliferation.

Beyond these canonical mechanisms, HPV infection induces extensive epigenetic remodeling, alters microRNA expression profiles, and reshapes the tumor immune microenvironment. In many HPV-positive tumors, viral integration and sustained oncoprotein expression are associated with enhanced antigen presentation, increased immune cell infiltration, and distinct inflammatory signaling patterns compared with HPV-negative tumors (13). These biological features contribute to the unique clinical behavior of HPV-driven cancers, including improved prognosis and greater sensitivity to radiotherapy and systemic therapies. Understanding these mechanisms is essential for interpreting the distinct epidemiological and clinical patterns observed between HPV-associated and HPV-negative oral and OP SCC.

In contrast to OP SCC, the role of HPV in OC SCC remains far less clear. Although HPV DNA and altered in p16(INK4a) expression have been detected in subsets of OC SCC, multiple studies indicate that HPV positivity does not consistently translate into prognostic benefit or establish a clear etiological role. Survival advantages associated with HPV appear to be largely restricted to OP tumors, whereas OC SCC continues to be predominantly driven by established carcinogenic exposures, including tobacco use, alcohol consumption, and local environmental factors (1, 15–17). These observations underscore the importance of avoiding direct extrapolation of OP SCC-derived biological and clinical paradigms to OC malignancies without appropriate anatomical and molecular stratification (18).

### 1.3 HPV genotypes and oncogenic potential

Human papillomaviruses (HPVs) are broadly classified into low-risk (LR) and high-risk (HR) genotypes according to their oncogenic potential. LR types, such as HPV6, HPV11, are primarily associated with benign epithelial proliferative lesions like condylomas and papilloma. In contrast, HR genotypes, including HPV16, HPV18, HPV31, HPV33, HPV35, HPV45, HPV52, and HPV81, have been implicated in the development of malignant lesions and are recognized etiological agents in several human cancers, including HNSCC (19). Among these, HPV16 is by far the most prevalent genotype detected in HNSCC, particularly in OP SCC, accounting for the vast majority of HPV-driven tumors worldwide. Although less frequently detected, other HR genotypes have also been reported in OC and OP carcinomas and may contribute to regional, demographic, and clinicopathological variability. Emerging evidence suggests that genotype-specific differences may influence viral persistence, tumor biology, and clinical outcomes, although these associations remain incompletely understood.

The predominance of HPV16, together with the persistence of other vaccine-covered HR genotypes, underscores the importance of HPV genotype surveillance for cancer prevention, epidemiological monitoring, and patient management. Furthermore, understanding genotype distribution is essential for evaluating the potential impact of current prophylactic vaccination programs on the future burden of HPV-associated oral and oropharyngeal cancers (17, 20).

### 1.4 Oncogenic viruses in head and neck carcinogenesis

Beyond HPV, several other oncogenic viruses have been proposed as potential contributors to head and neck carcinogenesis owing to their oncogenic properties, ability to establish persistent infections, and capacity to modulate host immune responses. Among these, Epstein-Barr virus (EBV), herpesviruses, and polyomaviruses have been investigated in OC and OP SCC, although considerably less extensively than HPV (21–25). To date, the available evidence remains limited, heterogeneous, and largely descriptive, with no consistent demonstration of causality or prognostic significance comparable to that observed for HPV. Furthermore, substantial variability in detection methods, tissue sampling strategies, and diagnostic criteria complicates interpretation and limits comparability across studies (15).

Given the extensive literature on HPV and its well-established etiological role in OP SCC, contrasted with the ongoing uncertainty surrounding its relevance in OC SCC, a comprehensive and systematic synthesis of the available evidence is warranted. Moreover, most previous systematic reviews have focused predominantly on HPV-positive OP SCC, with limited direct comparison to OC tumors and minimal evaluation of non-HPV oncogenic viruses. Therefore, the present systematic review aims to critically evaluate the prevalence, detection methods, clinicopathological associations, and prognostic impact of oncogenic viruses in OC and OP SCC. Emphasis is placed on site-specific viral contributions, the integration of evidence across multiple oncogenic viruses, methodological heterogeneity, and the identification of key knowledge gaps that warrant further research.

## 2 Methods

### 2.1 Study design and reporting standards

**Figure 1:**
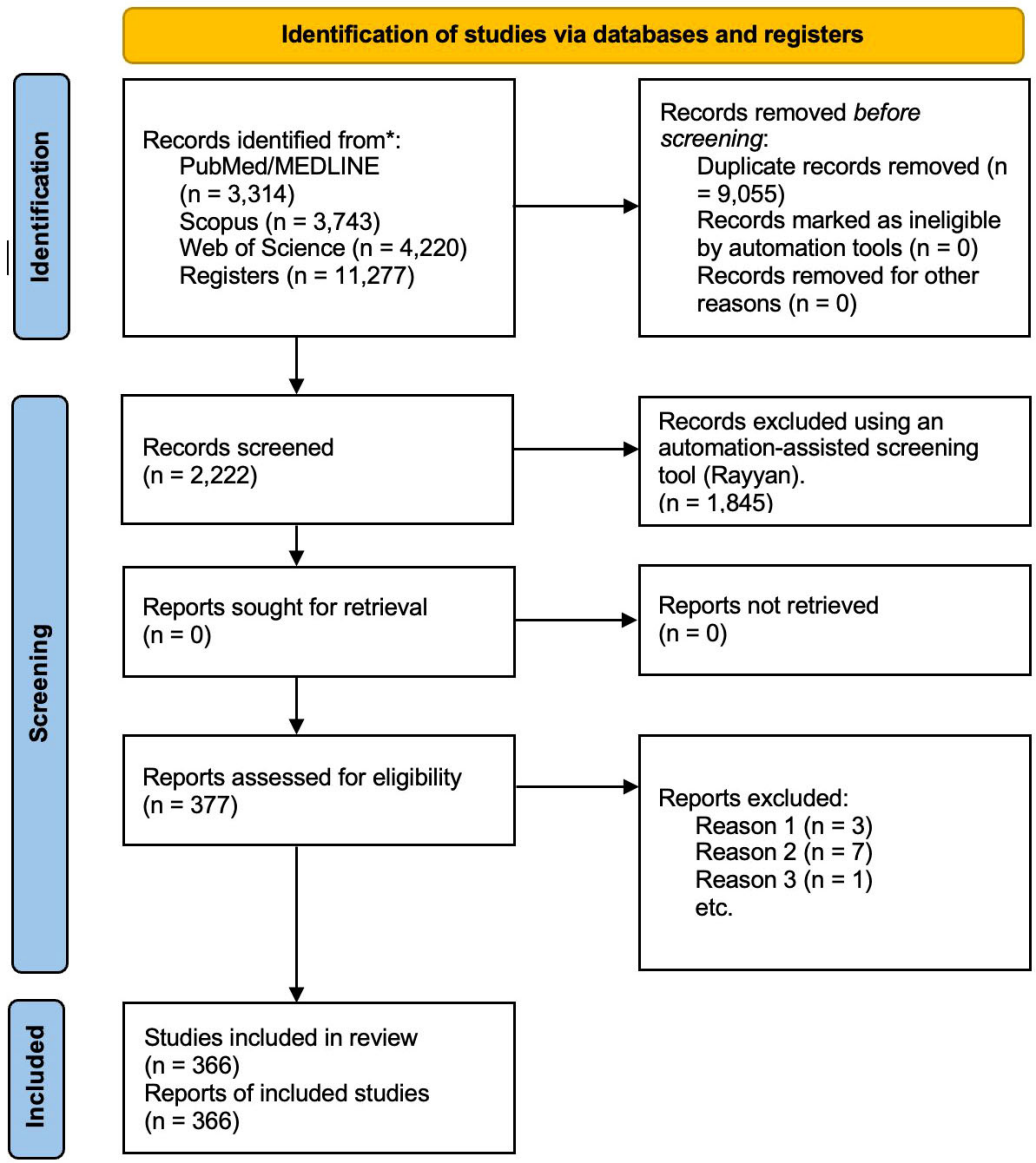
PRISMA 2020 flow diagram of the study selection process, including identification, screening, eligibility assessment, and final inclusion of studies. Records were identified through database searches in PubMed/MEDLINE (n = 3,314), Scopus (n = 3,743), and Web of Science (n = 4,220). After duplicate removal (n = 9,055), 2,222 records were screened by title and abstract using an automation-assisted tool (Rayyan), of which 1,845 were excluded. A total of 377 full-text articles were assessed for eligibility, and 11 were excluded due to non-relevant anatomical site, non-squamous histology, lack of viral assessment in tumor tissue, insufficient methodological detail, unclear classification, or overlapping cohorts. Ultimately, 366 studies were included in the qualitative synthesis.

This systematic review was conducted in accordance with the Preferred Reporting items for Systematic Reviews and Meta Analyses (PRISMA) 2020 guidelines (**Figure 1**). The methodology was defined *a priori* to ensure transparency, methodological rigor, and reproducibility throughout all stages of the review process.

### 2.2 Eligibility criteria

Eligibility criteria were established *a priori* using the Population, Exposure, Comparator, Outcomes, and Study design (PECOS) framework.

### 2.3 Population

Studies including human participants diagnosed with OC SCC and/or OP SCC were eligible. Diagnosis had to be confirmed by histopathological examination or standardized clinical pathological criteria.

### 2.4 Exposure

The exposure of interest was infection with oncogenic viruses, including HPV, EBV, HSV, and other viruses with established oncogenic potential. Viral presence or activity had to be directly assessed in tumor tissue using validated detection methods, such as polymerase chain reaction (PCR) or quantitative PCR (qPCR), immunohistochemistry (IHC), *in situ* hybridization (ISH), or sequencing based techniques.

### 2.5 Comparator

When applicable, comparators included virus negative tumor samples, non-tumor oral or oropharyngeal tissues, or control populations without OC or OP SCC. Studies without explicit comparators were included if they reported relevant descriptive, clinical, or mechanistic outcomes.

### 2.6 Outcomes

Eligible studies reported at least one of the following outcomes: association between viral infection and OC or OP SCC development; clinicopathological characteristics, including tumor stage, grade, or anatomical site; prognosis outcomes such as survival or treatment response; or molecular mechanisms involved in virus associated carcinogenesis.

### 2.7 Study design and report characteristics

Eligible studies included observational studies (cohort, case control, and cross sectional), retrospective or prospective clinical studies, and translational or pathological studies using human samples. Only original research articles published in peer reviewed journals between January 2015 and December 2025, including early online publications later assigned to a 2026 issue, and written in English, were considered. Reviews, meta-analyses, editorials, letters, conference proceedings, book chapters, these, and other non-original publication types were excluded through database level filters when available and through title/abstract screening when such filters were insufficient or inconsistent across databases. Studies were additionally excluded if they were conducted exclusively *in vitro* or in animal models without validation in human tissue; focused on head and neck cancers other than squamous cell carcinoma; did not clearly classify anatomical sites as oral cavity or oropharynx; or did not directly evaluate viral exposure in tumor tissue. Studies using non-validated methods or providing insufficient methodological detail were also excluded.

### 2.8 Information sources

A comprehensive literature search was performed in PubMed/MEDLINE, Scopus, and Web of Science Core Collection. In addition, the reference lists of included studies and relevant reviews were manually screened to identify additional eligible articles. The final search was conducted on January 26, 2026.

### 2.9 Search strategy

Search strategies were developed using a combination of controlled vocabulary terms (e.g. MeSH in PubMed) and free text keywords related to oral and oropharyngeal squamous cell carcinoma and oncogenic viruses. The complete PubMed/MEDLINE search strategy is provided in the Supplementary Materials and was adapted for Scopus and Web of Science using database specific syntax and filters. Database level filters were applied to restrict results to original research articles, as well as to predefined language (English) and publication date limits (2015–2025), in accordance with the eligibility criteria.

### 2.10 Study selection

All retrieved records were imported into Rayyan for deduplication and screening. Two reviewers independently screened titles and abstracts for eligibility, followed by full text assessment of potentially relevant studies. Disagreements were resolved through discussion and consensus.

### 2.11 Data extraction

Data were independently extracted by two reviewers using a standardized extraction form. Extracted variables included author, year of publication, country, study design, sample size, anatomical site, viral detection method, viral type or subtype, and reported clinical, pathological, or molecular outcomes. Discrepancies were resolved by consensus.

### 2.12 Data synthesis

Due to heterogeneity in study designs, viral detection methods, and reported outcomes, a qualitative narrative synthesis was conducted. Findings were synthesized according to virus type and anatomical site (OC SCC versus OP SCC). No meta-analyses were performed.

### 2.13 Reporting bias and certainty of evidence

A formal risk of bias assessment tool was not applied due to heterogeneity; However, key sources of bias were qualitatively evaluated. This represents a limitation of the present review and should be considered when interpreting the findings.

### 2.14 Registration

This review was not registered in PROSPERO due to its broad scope encompassing multiple oncogenic viruses and heterogeneous study designs, which limited the feasibility of a predefined quantitative synthesis.

## 3 Results and discussion

### 3.1 Study selection

The literature search identified 11,277 records across electronic databases, including PubMed/MEDLINE (n = 3,314), Scopus (n = 3,743), and Web of Science (n = 4,220). After removal of duplicates, 2,222 records remained and were screened based on titles and abstracts. During this stage, 1,845 records were excluded because they did not meet the predefined eligibility criteria, primarily due to irrelevant anatomical sites, non-squamous histology, absence of oncogenic viral assessment in tumor tissue, use of non-human study models, or publication type (reviews, editorials, and case reports). A total of 377 articles underwent full-text assessment for eligibility. Following full-text review, 11 studies were excluded due to lack of confirmed OC or OP SCC diagnosis, absence of direct viral assessment in tumor tissue, insufficient methodological detail, unclear anatomical classification, or overlapping patient cohorts without novel data. Ultimately, 366 studies met all eligibility criteria and were included in the qualitative synthesis (**Figure 1**; **Supplementary Materials 1**–**3**).

### 3.2 General characteristics of included studies

The 366 studies included in this review were conducted across multiple geographic regions, although most originated from North America, Europe, and East Asia. The majority employed observational designs, including case-control, cohort, and cross-sectional approaches, while a smaller proportion consisted of retrospective clinical or pathological investigations. Sample sizes varied considerably, ranging from 16 to 23,456 patients, highlighting substantial heterogeneity in study scale, design, and methodological approaches.

### 3.3 Geographic distribution and epidemiological patterns of HPV-associated disease in OC and OP SCC

Across the 366 included studies, a clear geographic imbalance was observed, with most studies originating from North America, Europe, and East Asia, whereas regions such as Africa and Latin America were comparatively underrepresented. Sample sizes varied substantially, ranging from small single-center cohorts (<100 patients) to large population-based datasets exceeding 10,000 cases. Studies from high-income regions generally reported higher HPV prevalence in OP SCC, whereas studies from low- and middle-income settings demonstrated greater variability, likely reflecting differences in risk factor profiles, healthcare access, and diagnostic infrastructure.

A structured synthesis of demographic and epidemiological variables revealed consistent patterns associated with HPV status, particularly in OP SCC. Across multiple cohorts, HPV-positive OP SCC occurred more frequently in younger patients, typically within the fifth to sixth decades of life, compared with HPV-negative tumors (26). Although males predominated across both OC SCC and OP SCC, several studies reported a relatively higher proportion of females among HPV-positive OP SCC cases, suggesting potential differences in exposure patterns or biological susceptibility. Behavioral risk factors further distinguished HPV-driven tumors from non-viral carcinogenesis, as HPV-positive OP SCC was consistently associated with lower cumulative tobacco exposure and reduced alcohol consumption compared with HPV-negative disease.

Large population-based studies from North America and Europe indicate that HPV-positive tumors now account for a substantial proportion of OP SCC cases, frequently exceeding 50-60%, and are characterized by younger age at diagnosis alongside marked racial, socioeconomic, and regional disparities (27–30). In the United States, HPV positivity is strongly influenced by race, socioeconomic status, and geographic region, whereas Northern European cohorts have demonstrated a pronounced temporal increase in OP SCC incidence driven predominantly by HPV-positive disease (27, 29, 31).

Outside high-income Western regions, HPV-positive OP SCC has historically exhibited lower prevalence but is increasingly reported (32). Studies from Eastern China, Malaysia, and Brazil have documented HPV positivity rates ranging from approximately 30% to 60%, accompanied by demographic shifts toward younger age, female sex, and reduced exposure to tobacco and alcohol (33–35). In contrast, data from sub-Saharan Africa indicate that HPV remains a relatively minor etiological contributor to OP SCC, highlighting substantial global variability in viral-attributable risk and reflecting differences in population structure, exposure patterns, and diagnostic infrastructure (36, 37).

Across geographic regions, HPV-positive OP SCC displays a reproducible epidemiological and anatomical profile, including preferential involvement of the tonsil and base of tongue, lower prevalence of traditional carcinogenic exposures, and superior survival outcomes compared with HPV-negative disease (38–41). However, the contribution of HPV to the overall OP SCC burden is not uniform. Some populations demonstrate stable proportions of HPV-positive tumors despite rising OP SCC incidence, suggesting the coexistence of additional etiological drivers beyond viral infection (42).

Several studies also reported clinically relevant epidemiological implications. HPV-positive OP SCC frequently presents with occult primary tumors or isolated cervical lymphadenopathy, potentially contributing to diagnostic delay (43, 44). Nevertheless, prognosis remains favorable overall, although outcomes vary according to viral genotype, disease stage, and healthcare context (39, 45). HPV genotype distribution is dominated by HPV16, with most high-risk genotypes covered by current vaccines, reinforcing the population-level relevance of vaccination strategies for disease prevention (46, 47). Epidemiological evidence further highlights broader public health considerations, including potential transmission dynamics among partners and the need for region-specific prevention, surveillance, and screening strategies (48–50). Collectively, these findings position HPV-positive OP SCC as a growing global cancer burden characterized by substantial demographic and geographic heterogeneity, emphasizing the importance of tailored public health interventions and standardized surveillance frameworks (51–53).

In contrast, the epidemiological profile of OC SCC differs markedly from that of OP SCC. Across retrospective and cross-sectional cohorts, HPV prevalence in OC SCC was generally low, ranging between 7% and 10%, and HPV status did not consistently emerge as an independent prognostic factor for overall or disease-specific survival (54, 55). Evidence from high-incidence regions further reinforces the dominant role of established carcinogenic exposures in OC SCC. In several cohorts, HPV-positive or p16(INK4a)-positive tumors occurred predominantly among patients with substantial tobacco exposure, arguing against HPV as an independent risk factor and highlighting the strong confounding influence of traditional behavioral risks (56, 57). Consistent with this interpretation, molecular studies have demonstrated minimal transcriptionally active HPV in OC SCC, suggesting that viral detection often reflects incidental infection rather than biologically meaningful oncogenic involvement (58).

Although geographic variability in HPV DNA detection has been reported in Latin American and European cohorts, these findings have not translated into reproducible associations with malignant transformation, prognosis, or clinical outcomes. Likewise, HPV detection in benign or potentially malignant oral lesions has not been linked to subsequent OC SCC development, indicating that HPV is unlikely to function as an early oncogenic event in oral carcinogenesis (19, 59). Similarly, while moderate HPV positivity rates have been reported in mixed head and neck cancer cohorts, site-specific analyses do not support a causal role for HPV in OC SCC (60).

Demographic differences, including higher p16(INK4a) expression among younger patients and females, have occasionally been reported; however, these observations do not establish a direct viral etiology and may instead reflect underlying biological heterogeneity or differences in exposure profiles (61, 62). Importantly, integrative risk models indicate that OC SCC development is best explained by multifactorial interactions, in which HPV likely acts as a secondary or synergistic factor in the context of tobacco exposure and host genetic susceptibility, particularly alterations in p53 (63).

Taken together, the epidemiological evidence supports two distinct disease paradigms. HPV-positive OP SCC exhibits a well-defined demographic, clinical, and geographical profile characterized by younger age at diagnosis, reduced exposure to traditional behavioral risk factors, and favorable clinical outcomes. In contrast, OC SCC remains primarily associated with long-term carcinogen exposure, with HPV playing at most a limited and context-dependent role. The consistency of these observations across diverse geographic regions and study designs underscores the importance of site-specific interpretation of HPV-related carcinogenesis and cautions against extrapolating OP SCC-based paradigms to OC malignancies (19, 54, 56, 58, 60, 64–66).

### 3.4 Anatomical distribution and evidence for oncogenic viruses in OC and OP SCC

Of the 366 included studies, 96 focused exclusively on OC SCC, 150 examined OP SCC, and 120 included both anatomical sites. Studies investigating viral oncogenesis were more frequently conducted in OP SCC, particularly those evaluating HPV. Among OC SCC studies, the tongue, gingiva, and floor of the mouth were the most frequently reported anatomical subsites when specified. In contrast, OP SCC studies predominantly involved tumors of the tonsillar region and base of tongue, anatomical locations that consistently demonstrated the strongest association with HPV infection.

HPV was by far the most frequently investigated oncogenic virus, being evaluated in 319 studies. This predominance reflects both the extensive body of evidence supporting its role in head and neck carcinogenesis and the recognition of HPV-positive OP SCC as a distinct biological and clinical entity. Although several HR genotypes, including HPV18, HPV31, HPV33, HPV35, HPV45, HPV52, and HPV58, were reported, HPV16 consistently accounted for the vast majority of HPV-positive OP SCC cases across geographic regions (67, 68). While genotype-specific analyses were inconsistently reported, the predominance of HPV16 was one of the most reproducible findings across studies, reinforcing its central role in HPV-driven carcinogenesis. Emerging evidence further suggests that specific HPV genotypes may differ in their epidemiological distribution, biological behavior, and prognostic implications, although these associations require further validation.

Multiple lines of evidence support HPV as the dominant oncogenic virus in OP SCC. HPV-positive tumors frequently present with cervical lymphadenopathy rather than primary mucosal symptoms, reflecting a distinct disease biology that may contribute to diagnostic delay while underscoring the virus-driven nature of these malignancies (69). Longitudinal analyses have demonstrated a marked increase in the portion of HPV-positive tonsillar SCC over time, while genotype-specific studies suggest potential prognostic differences between HPV16- and HPV18-associated tumors (70, 71). Moreover, even after distant metastasis, HPV-positive OP SCC generally exhibits prolonged survival compared with HPV-negative disease, although host-related factors such as smoking status and metastatic burden continue to influence outcomes (72).

**Figure 2:**
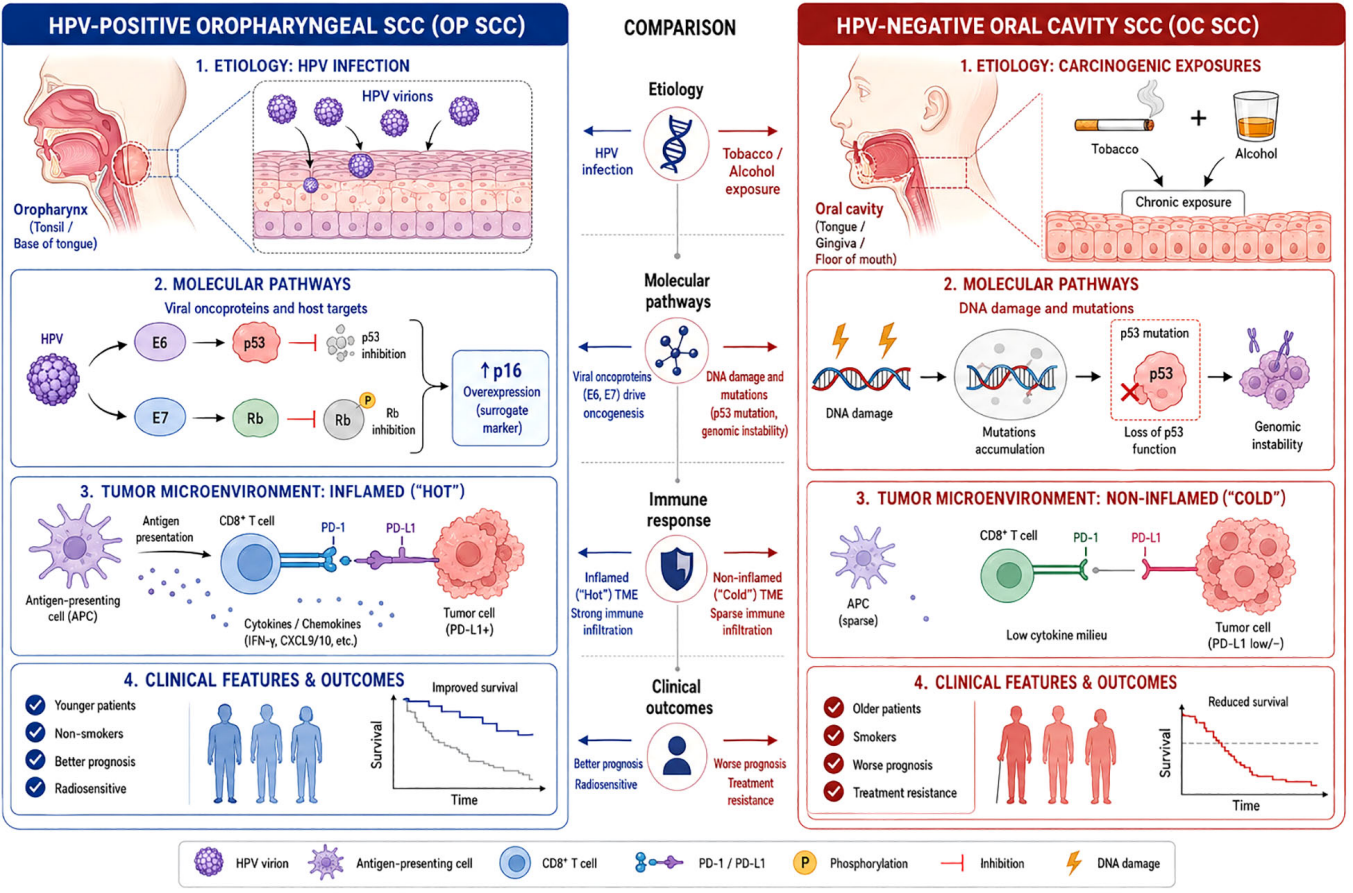
Schematic overview of the biological and clinical differences between HPV-positive oropharyngeal (OP) and HPV-negative oral cavity (OC) squamous cell carcinoma (SCC). HPV-driven OP SCC is characterized by viral oncoproteins activity (E6/E7), p53 degradation, Rb pathway inhibition, and p16(INK4a) overexpression, along with an inflamed tumor microenvironment (TME) marked by increased immune cell infiltration. In contrast, HPV-negative OC SCC is primarily associated with carcinogen exposure (e.g., tobacco and alcohol), leading to genomic instability, frequent TP53 mutations, and absence of viral elements. This subtype typically exhibits a “cold” TME, characterized by low immune cell infiltration, limited immune activation, and an immunosuppressive profile, which are associated with unfavorable clinical outcomes and reduced therapeutic responsiveness. These differences translate into distinct clinical outcomes, with HPV-positive OP SCC exhibiting improved prognosis and greater radiosensitivity, whereas HPV-negative OC SCC is associated with poorer prognosis and increased treatment resistance. This illustration was generated using ChatGPT (OpenAI, GPT-4 version, May 2026). The resulting image was reviewed by the authors to ensure scientific accuracy.

In contrast, studies focusing on OC SCC consistently report low prevalence of HR HPV DNA, even when highly sensitive molecular techniques are applied (73). Large cohort studies from Brazil, and the United Kingdom documented HPV detection rates below 4% or complete absence of HPV in OC SCC, supporting a negligible etiological role for HPV in OC carcinogenesis compared with traditional risk factors such as tobacco and alcohol use (74, 75). Population-specific studies further emphasize regional differences in HPV attribution. In several Asian cohorts, discordance between p16(INK4a) expression and HPV DNA status was frequent, reducing the reliability of p16(INK4a) as a surrogate marker and highlighting that true HPV-driven OP SCC may represents a smaller subset than that observed in Western populations (33, 76). These findings underscore the importance of molecular confirmation when attributing oncogenesis to HPV, particularly outside high-prevalence regions. These differences are summarized in **Figure 2**.

Although emerging evidence from rare populations, including pediatric OC SCC, suggests that HPV may contribute to tumorigenesis in select contexts, limited case numbers and heterogeneous methodologies preclude definitive conclusions (77). Overall, the available evidence reinforces the biological and clinical distinction between HPV-positive OP SCC and HPV-negative disease, with important implications for risk stratification, screening strategies, and therapeutic decision-making.

The clinical relevance of HPV-positive tumors is further supported by their distinct biological characteristics. Compared with HPV-negative disease, HPV-driven tumors exhibit increased immune cell infiltration, enhanced antigen presentation, and elevated expression of immune checkpoint molecules, including programmed cell death protein 1 (PD-1) and programmed death-ligand 1 (PD-L1), suggesting potential implications for immunotherapy responsiveness (78). In contrast, HPV-negative tumors, particularly those associated with tobacco exposure, frequently display a more immunosuppressive microenvironment characterized by genomic instability and reduced immune infiltration. These observations further support the concept that HPV-positive and HPV-negative tumors represent biologically distinct disease entities.

In contrast to HPV, other oncogenic viruses were investigated far less frequently and demonstrated substantially weaker evidence for a causal role in either OC SCC or OP SCC. EBV was evaluated in 32 studies, primarily in OC SCC cohorts, whereas HSV, MCPyV, and CMV were investigated in only 5, 3, and 1 study, respectively. These viruses were generally assessed in small cohorts and often using single-method detection approaches, limiting the strength and reproducibility of the available evidence (23, 24, 79, 80).

Among non-HPV viruses, EBV received the greatest attention; however, reported prevalence rates, biological relevance, and clinical associations varied substantially across studies. Several investigations documented EBV positivity in OC SCC tissues and suggested potential associations with tumor differentiation, immune modulation, inflammatory signaling, elevated systemic IL-10 levels or tobacco exposure, raising the possibility that EBV may interact with established environmental carcinogens rather than function as an independent oncogenic driver (81, 82).

Conversely, multiple case-control and seroepidemiological studies failed to demonstrate a clear etiological role for EBV in OC SCC. High EBV immunoglobulin G (IgG) seropositivity was observed in both OC SCC patients and healthy controls without evidence of active infection, while EBV DNA was detected at comparable frequencies in malignant and non-malignant oral tissues, with no consistent associations with tumor grade, sex, or disease status (83). Geographic variability further complicated interpretation. Although relatively high EBV DNA prevalence has been reported in certain Eastern European cohorts, these findings have not been conclusively linked to OC SCC pathogenesis. In contrast, studies from Iran and Southeast Asia consistently reported low EBV detection rates and a lack of significant associations with oral cancer development (83–85).

The role of EBV in OP SCC appears even more limited. Large cohort studies evaluating both HPV and EBV consistently demonstrated low EBV prevalence and rare co-infection events, with HPV emerging as the dominant viral determinant of tumor behavior and prognosis (86–88). Although potential synergistic interactions between HPV and EBV have been proposed, evidence supporting a clinically meaningful co-infection model remains preliminary. Occasional HPV/EBV co-infections have been reported, but these cases have not been consistently associated with distinct clinicopathological features or prognostic outcomes. More recent molecular studies suggest that co-infection may influence microRNA expression, DNA methylation profiles, or inflammatory signaling pathways; however, these observations remain largely mechanistic and have yet to translate into reproducible clinical correlations (89–92).

Evidence implicating other oncogenic viruses, including HSV, MCPyV, and CMV, remains extremely limited. Available studies are predominantly descriptive, involve small sample sizes, and have not demonstrated consistent associations with tumor initiation, progression, prognosis, or treatment response. Even when viral nucleic acids are detectable within tumor tissues, their biological significance remains uncertain.

Taken together, the available evidence demonstrates a marked disparity in both study volume and methodological rigor across oncogenic viruses. HPV-related evidence is derived from a substantially larger number of studies, frequently incorporates multimodal detection approaches, and consistently supports a causal, biological, and prognostic role in OP SCC. In contrast, evidence for EBV, HSV, MCPyV, CMV, and other non-HPV viruses remains limited, heterogeneous, and often inconclusive. Consequently, current data support HPV as the only oncogenic virus with a reproducible and clinically relevant role in OC and OP SCC, particularly within the oropharynx, whereas the contribution of other viruses remains uncertain and requires further investigation.

### 3.5 Biological basis of HPV-driven carcinogenesis

The epidemiological, clinicopathological, and prognostic differences observed between HPV-positive and HPV-negative tumors are supported by distinct molecular mechanisms underlying HPV-driven carcinogenesis. Evidence derived from molecular, transcriptomic, and immunological studies included in this review indicates that HPV-associated SCC represent biologically distinct disease entities characterized by viral oncogene activity, immune modulation, epigenetic remodeling, and tumor microenvironment reprogramming (93).

A central feature of HPV-driven carcinogenesis is the disruption of cell-cycle regulation through viral oncogenes rather than through the accumulation of canonical tumor suppressor gene mutations. HPV-positive tumors frequently retain wild-type TP53 but exhibit functional inactivation of the p53 pathway through E6-mediated degradation, accompanied by dysregulation of p16(INK4a) expression and pRB signaling (94, 95). These mechanisms contribute to a molecular profile fundamentally different from that observed in HPV-negative tumors.

Despite sharing common viral drivers, HPV-positive cancers exhibit substantial molecular heterogeneity. Large-scale genomic and transcriptomic analyses have identified distinct molecular subtypes characterized by differences in immune activation, epithelial differentiation, viral integration patterns, and somatic alterations (96, 97). Alterations in TRAF3 and CYLD have been associated with episomal HPV maintenance, NF-κB pathway activation, and improved survival outcomes, suggesting the existence of alternative oncogenic pathways within HPV-positive tumors (96). Furthermore, differential viral integration into cancer associated genes has been linked to tumor persistence and recurrence, indicating that integration patterns may influence clinical behavior (13).

Epigenetic remodeling also appears to play a significant role in HPV-mediated tumorigenesis. Studies have reported HPV-associated promoter methylation of tumor suppressor genes, altered expression of DNA damage-response regulators, and widespread changes in chromatin-associated pathways (98). Likewise, virus-associated alterations in DNA methylation and histone modification profiles affect genes involved in cell-cycle regulation, differentiation, and immune surveillance, thereby contributing to malignant progression (99). In contrast, dysregulation of H2AX and γ-H2AX pathways has been more strongly associated with HPV-negative tumors, supporting distinct mechanisms of genomic instability between viral and non-viral carcinogenesis (100).

Another recurrent feature is the deregulation of non-coding RNAs. Multiple studies identified differential expression of miR-363, miR-21, miR-155, and other regulatory microRNAs associated with HPV status, smoking behavior, and survival outcomes (101, 102). These findings suggest that microRNAs act as important intermediaries through which viral infection reshapes host gene expression networks.

The tumor microenvironment is also profoundly influenced by viral status. Transcriptomic and proteomic analyses consistently demonstrate that HPV-positive tumors exhibit increased adaptive immune infiltration, enhanced antigen presentation, Th1-skewed cytokine profiles, and distinct inflammatory signaling patterns compared with HPV-negative tumors, which are frequently characterized by fibroblast enrichment, extracellular matrix remodeling, and immunosuppressive features (103, 104). These immune differences may partially explain the superior prognosis and enhanced treatment responsiveness commonly observed in HPV-positive OP carcinomas.

Recent immunogenomic studies have further expanded the understanding of viral antigenicity. Beyond the canonical E6 and E7 oncoproteins, HPV E1 and E2 proteins have been shown to elicit robust cytotoxic T-cell responses, highlighting additional targets for antigen-directed immunotherapies and therapeutic vaccine development (14, 20).

In contrast, evidence supporting comparable molecular mechanisms for non-HPV oncogenic viruses remains limited. Studies investigating EBV and other viruses have reported variable molecular alterations, inconsistent transcriptional activity, and limited evidence of biologically relevant viral integration, precluding definitive conclusions regarding causality (105). Consequently, a coherent molecular framework comparable to that established for HPV-driven carcinogenesis is currently lacking for other oncogenic viruses associated with OC SCC and OP SCC.

Collectively, these findings provide a biological rationale for the distinct clinical behavior of HPV-positive tumors and reinforce the concept that HPV-driven carcinogenesis involves a complex interplay among viral oncogene activity, epigenetic remodeling, immune regulation, and tumor microenvironmental reprogramming. At the same time, the observed molecular heterogeneity highlights the need for further high-resolution molecular studies to refine biological stratification and support the development of precision therapeutic strategies.

### 3.6 Clinicopathological associations, prognosis and treatment response

Multiple studies reported significant associations between oncogenic viral infection and the clinicopathological characteristics of OC SCC and OP SCC (106, 107). Among all investigated viruses, HPV demonstrated the strongest and most consistent associations with distinct tumor features. HPV-positive tumors were predominantly associated with the oropharynx, particularly the tonsillar region and base of tongue, and exhibited clinicopathological profiles that differed substantially from those of virus-negative tumors. In contrast, associations between non-HPV oncogenic viruses and pathological parameters such as tumor stage, grade, or anatomical distribution were inconsistent across studies. Although EBV positivity was occasionally linked to specific clinicopathological characteristics, findings varied considerably between cohorts and no reproducible pattern emerged. Overall, the evidence supporting clinicopathological associations was substantially stronger for HPV than for any other oncogenic virus.

These clinicopathological differences were accompanied by marked differences in patient outcomes. Across multiple cohorts, HPV-positive OP SCC was consistently associated with improved overall survival and disease-free survival compared with HPV-negative disease. This favorable prognostic profile represents one of the most reproducible findings identified across the included studies and further supports the classification of HPV-positive OP SCC as a distinct biological and clinical entity. In contrast, the prognostic significance of viral infection in OC SCC remained considerably less clear. Studies evaluating HPV in OC SCC, as well as investigations involving EBV, HSV, MCPyV, and other oncogenic viruses, produced heterogeneous results and failed to demonstrate consistent associations with either improved or worsened survival outcomes.

The prognostic advantage observed in HPV-positive OP SCC was accompanied by differences in treatment response. Although not uniformly reported across all studies, multiple cohorts demonstrated enhanced response to chemoradiotherapy and increased radiosensitivity among HPV-positive tumors compared with HPV-negative disease. These findings support the role of HPV status as a clinically relevant predictive biomarker and have contributed to the development of risk-adapted treatment strategies for patients with OP SCC. Mechanistically, improved therapeutic responsiveness has been associated with preserved apoptotic pathways, lower mutational burden, enhanced immune activation, and a more immunologically active tumor microenvironment, all of which may contribute to increased treatment sensitivity and superior clinical outcomes.

Conversely, evidence regarding treatment response in OC SCC remained limited and inconsistent. Available studies did not provide clear support for a predictive role of HPV in oral cavity tumors, and investigations evaluating EBV or other non-HPV oncogenic viruses similarly failed to demonstrate reproducible associations with treatment outcomes. Consequently, current evidence does not support the routine use of non-HPV viral status as a biomarker for prognostic assessment or therapeutic stratification in either OC SCC or OP SCC.

Taken together, the available data indicate that HPV-positive OP SCC is characterized by a distinctive clinicopathological profile, favorable prognosis, and enhanced responsiveness to treatment, reinforcing its status as a biologically unique disease entity. In contrast, the clinical significance of HPV in OC SCC and the role of other oncogenic viruses remain uncertain, with insufficient evidence to establish consistent prognostic or predictive value.

### 3.7 Methodological patterns and sources of heterogeneity

A major finding of this systematic review was the substantial methodological heterogeneity across studies, which significantly influenced the interpretation of viral prevalence, clinicopathological associations, prognostic implications, and treatment-related outcomes in OC SCC and OP SCC. Variability was observed in study design, sample size, specimen type, viral detection methods, analytical platforms, and criteria used to define viral positivity, all of which contributed to differences in reported findings and limited direct comparability across studies.

A wide range of viral detection methods was employed among the included studies. PCR and qPCR were the most frequently used techniques, either alone or in combination with other approaches. IHC and ISH were also commonly applied, particularly in studies evaluating HPV status in OP SCC. Several investigations employed combined molecular and immunohistochemical approaches to improve diagnostic accuracy, whereas viral genotyping, when reported, focused predominantly on HPV16, with limited and inconsistently reported data for other viral genotypes. This variability in detection methods and reporting standards represented an important source of heterogeneity across the literature.

To better quantify the sources of heterogeneity, a structured methodological assessment was performed. Sample sizes ranged from small mechanistic cohorts (<100 patients) to large population-based studies exceeding 1,000 patients, with many studies falling within the intermediate range of 100–500 patients. Of the 366 included studies, methodological information regarding viral detection techniques was available for 341 studies, whereas 25 studies reported insufficient or missing methodological data and were excluded from method-specific analyses.

Among studies with available methodological information, PCR-based approaches were the most frequently used detection methods (61%), followed by IHC (60.7%). ISH and viral genotyping were reported in 19% and 16.1% of studies, respectively. Because many studies employed more than one detection method, these percentages were not mutually exclusive. To further characterize diagnostic strategies, studies were further categorized according to the number of methods used. Single-method approaches accounted for 54% of studies, dual-method strategies for 34%, and multimodal approaches for only 12%. Despite the widespread use of PCR and IHC, relatively few studies employed multimodal strategies, which are generally considered more robust for identifying transcriptionally active viral infection. A detailed comparison of detection strategies and their methodological implications is presented in **Table 1**.

**Table 1:** Distribution of viral detection methods and their impact on HPV prevalence and interpretation across included studies.

Detection strategy	Studies (n, %)	Technical basis	Strengths	Limitations	Impact on HPV prevalence reporting	Interpretation
PCR	208 (61%)	Detection of viral DNA	High sensitivity; widely available; allows genotyping	Cannot distinguish transcriptionally active infection	Moderate prevalence estimates; may include latent/passenger virus	Overestimation risk in OC SCC; acceptable in OP SCC when combined with clinical data
IHC	207 (60.7%)	Surrogate marker of HPV-driven oncogenesis	Cost-effective; easy to implement; prognostic relevance in OP SCC	Low specificity; false positives unrelated to HPV	Higher HPV prevalence reported compared to molecular methods	Reliable surrogate in OP SCC; limited specificity in OC SCC
ISH	65 (19%)	Localization of viral DNA/RNA in tumor cells	Higher specificity; confirms viral presence in tumor tissue	Lower sensitivity; technically demanding	Lower and more specific prevalence estimates	Supports causal inference mainly in OP SCC
Genotyping	55 (16.1%)	Identification of HPV subtypes	Provides epidemiological and prognostic insights	Does not confirm biological activity alone	Refines subtype distribution without altering prevalence significantly	Highlights HPV16 predominance in OP SCC
Dual	116 (34%)	Combined molecular and protein expression	Improved diagnostic accuracy; balances sensitivity and specificity	Still limited in confirming transcriptional activity	More conservative and reliable prevalence estimates	Recommended approach for OP SCC; reduces false positives in OC SCC
Multimodal (≥2 methods)	41 (12%)	Integrated molecular and histopathological assessment	Highest diagnostic accuracy; confirms biologically active infection	Resource-intensive; less frequently used	Most conservative and clinically relevant estimates	Gold standard for HPV-driven OP SCC; rarely applied in OC SCC

PCR, polymerase chain reaction; IHC, immunohistochemistry; ISH, *in situ* hybridization.

Importantly, methodological choice directly influenced reported HPV prevalence and its clinical interpretation. Studies relying on single-method approaches, particularly p16(INK4a) IHC alone, frequently reported higher HPV positivity rates, whereas multimodal strategies incorporating molecular confirmation produced more conservative and biologically meaningful estimates. This discrepancy was especially relevant in OC SCC, where HPV detection may reflect incidental viral presence rather than true oncogenic involvement. In contrast, studies evaluating OP SCC consistently demonstrated stronger evidence for a causal and prognostic role of HPV when combined molecular and immunohistochemical approaches were used.

The widespread use of p16(INK4a) as a surrogate marker for HPV infection introduced additional complexity. Although p16(INK4a) positivity correlates with several clinicopathological characteristics (108), molecular studies have demonstrated that p16(INK4a) overexpression does not uniformly indicate transcriptionally active HPV infection, resulting in biologically distinct subgroups with divergent clinical outcomes (109). Reliance on p16(INK4a) without confirmatory molecular testing therefore represents an important source of potential misclassification, particularly in OC SCC cohorts and in populations with lower HPV prevalence (110).

Additional methodological factors further complicated interpretation. Differences in tissue sampling procedures, specimen type, viral genotyping strategies, detection targets, and criteria used to define viral positivity all contributed to variability across studies. Non-invasive detection methods, including salivary RT-PCR, have demonstrated high diagnostic accuracy and clinical feasibility but often yield lower HPV positivity rates than tissue-based assays, highlighting the influence of sampling strategy on viral attribution (111). Similarly, quantitative molecular approaches assessing viral load or oncogene expression provide greater biological resolution but currently lack methodological standardization across studies (112).

Additional heterogeneity arose from the incorporation of non-viral prognostic biomarkers into multivariable models. Systemic inflammatory markers and host-related molecular factors, including estrogen receptor α expression, demonstrated prognostic relevance independent of viral status, emphasizing the multifactorial nature of disease behavior and complicating attribution of outcomes exclusively to viral infection (113, 114). Emerging technologies, including minimally invasive diagnostic platforms and high-throughput genotyping approaches, further diversify current diagnostic algorithms despite demonstrating promising accuracy and reproducibility (115, 116).

Geographic differences represented an additional source of heterogeneity. Larger cohort studies were predominantly conducted in North America and Europe, whereas studies from Asia and other regions more frequently consisted of smaller cohorts and demonstrated greater methodological variability (30, 41, 60, 117). A structured analysis of geographic distribution and sample size revealed marked regional disparities across the included studies (**Table 2**). North America and Europe contributed the largest number of studies and the highest cumulative patient volumes, largely driven by large-scale cohort investigations. In contrast, regions such as Africa, Southeast Asia, and Oceania remained underrepresented and were characterized by smaller sample sizes. Although East and South Asia contributed substantial numbers of patients, these data were frequently derived from heterogeneous study designs and variable detection methodologies. Consequently, global estimates of viral prevalence and clinical associations are disproportionately influenced by evidence generated in North America and Europe, while data from other regions remain comparatively limited.

**Table 2:** Geographic distribution and sample size characteristics of included studies.

Region	Number of studies (n)	Total patients (n)	Median sample size	Interpretation
North America	98	391,531	180.5	Largest contribution of large-scale cohort studies; strong representation in HPV-driven OP SCC research
Europe	107	42,524	139	High study density with moderate sample sizes; consistent HPV-related findings
East Asia	51	70,778	152	Substantial contribution with moderate-to-large cohorts; increased methodological variability
South Asia	39	420,452	98	High total patient count driven by large population-based datasets; heterogeneous methodologies
Latin America	21	17,529	82	Moderate representation; generally smaller cohorts and variable detection methods
Southeast Asia	16	2,533	100	Limited sample size; contributes to regional variability in HPV prevalence
West Asia	11	2,279	75	Small-to-moderate cohorts; underrepresented region
Africa	10	1,277	102.5	Limited data; small cohorts and scarce molecular studies
Oceania	6	685	86	Minimal representation; limited impact on global estimates
Mixed regions	5	3,527	432	Multiregional studies; limited comparability due to pooled populations
North Asia	2	105	52.5	Minimal representation; limited impact on global estimates

Additionally, incomplete reporting of methodological details, sample size, or viral detection criteria in a subset of studies further reduced reproducibility and limited cross-study comparisons. Collectively, these findings indicate that methodological heterogeneity remains one of the principal barriers to unified interpretation of the role of oncogenic viruses in OC SCC and OP SCC. Many discrepancies in reported viral prevalence, clinicopathological associations, and prognostic significance may reflect methodological variability rather than true biological differences.

Overall, the evidence highlights the urgent need for standardized diagnostic algorithms that integrate molecular confirmation with clinicopathological context, harmonized definitions of viral positivity, and broader adoption of multimodal detection strategies. Such standardization will be essential to improve comparability across studies, refine estimates of viral prevalence, and strengthen future investigations into the biological and clinical significance of oncogenic viruses in OC and OP SCC.

### 3.8 Strength of evidence for oncogenic viruses

To synthesize the heterogeneous evidence identified across anatomical sites and viral agents, the overall strength of evidence for each oncogenic virus was assessed based on the volume of available studies, methodological robustness, consistency of findings, biological plausibility, and reproducibility of clinicopathological and prognostic associations (**Table 3**).

**Table 3:** Strength of evidence for oncogenic viruses across oral cavity (OC) and oropharyngeal (OP) squamous cell carcinoma (SCC).

Oncogenic virus	OSCC-evidence strength	OPSCC-evidence strength	Type of supporting evidence	Overall interpretation
Human papillomavirus (HPV)	Low/Inconsistent	High/Consistent	Epidemiology, molecular (E6/E7), clinicopathology, prognosis	Causal and prognostic driver in OP SCC; limited role in OC SCC
Epstein-Barr virus (EBV)	Very low/Inconclusive	Very low/Inconclusive	molecular, serology, immunohistochemistry (IHC), small cohorts	Incidental detection; no consistent oncogenic role
Merkel cell polyomavirus (MCPyV)	Very low	Very low	Sporadic molecular-based studies	Descriptive only; no causal evidence
Cytomegalovirus (CMV)	Very low	Very low	Isolated epidemiological reports	No demonstrated oncogenic relevance
Herpes simplex virus (HSV)	Very low	Very low	Serology, limited molecular studies	No consistent association with carcinogenesis

Among all investigated viruses, HPV demonstrated the strongest and most consistent evidence, particularly in OP SCC. Across multiple geographic regions, study designs, and detection methodologies, HPV-positive OP SCC was reproducibly associated with distinct epidemiological characteristics, specific anatomical localization, unique molecular features, favorable clinical outcomes, and enhanced treatment responsiveness (38, 67, 89). Furthermore, substantial mechanistic evidence supports a direct oncogenic role for HPV through viral oncogene-mediated disruption of cell-cycle regulation, immune modulation, and tumor microenvironment reprogramming. Collectively, these findings support a strong level of evidence for HPV as an etiological, prognostic, and clinically relevant biomarker in OP SCC.

In contrast, the strength of evidence supporting HPV involvement in OC SCC was considerably weaker. Although HPV DNA has been detected in a subset of OC tumors, prevalence rates were generally low and highly variable across studies. Moreover, HPV positivity was not consistently associated with distinct clinicopathological characteristics, survival advantages, or treatment-related outcomes. Molecular studies further suggest that HPV detection in OC SCC may frequently represent incidental viral presence rather than biologically relevant oncogenic activity (15). Therefore, current evidence supports at most a limited and context-dependent role for HPV in OC carcinogenesis.

Among non-HPV oncogenic viruses, EBV represented the most extensively investigated agent; however, the available evidence remained inconsistent. Although EBV has been detected in subsets of OC SCC and OP SCC and occasional associations with inflammatory signaling, immune modulation, or specific clinicopathological characteristics have been reported, findings lacked reproducibility across populations and study designs (21, 82, 91). The absence of a consistent molecular framework, together with variable prevalence estimates and limited prognostic significance, precludes definitive conclusions regarding a causal or prognostic role for EBV in OC or OP carcinogenesis.

Evidence for HSV, MCPyV, CMV, and other oncogenic viruses was substantially more limited. Available studies were few, frequently involved small cohorts, and primarily reported descriptive epidemiological observations without demonstrating reproducible associations with tumor initiation, progression, prognosis, or treatment response (22, 25, 79). Consequently, the current evidence base is insufficient to support a clinically meaningful role for these viruses in OC SCC or OP SCC.

Importantly, the interpretation of evidence strength is influenced by substantial methodological heterogeneity across studies. Variability in viral detection methods, definitions of viral positivity, specimen types, and reporting standards contributes to discrepancies in prevalence estimates and clinical associations. Therefore, while the evidence supporting HPV-driven OP SCC remains robust and reproducible despite these limitations, conclusions regarding other oncogenic viruses should be interpreted cautiously until larger studies employing standardized diagnostic criteria become available.

Overall, the current body of evidence supports HPV as the only oncogenic virus with a well-established etiological and prognostic role in OP SCC. In contrast, evidence supporting HPV involvement in OC SCC remains limited, whereas the roles of EBV and other oncogenic viruses remain inconclusive. This comparative assessment highlights important differences in the strength of evidence across anatomical sites and viral agents and underscores the need for standardized methodologies and further high-quality investigations to clarify the contribution of non-HPV viruses to OC and OP carcinogenesis.

### 3.9 Current therapeutic approaches and clinical implications

The evidence synthesized in this review supports the growing recognition of HPV-positive and HPV-negative SCC as biologically distinct disease entities with important implications for clinical management. Current treatment strategies for OC SCC and OP SCC are primarily based on tumor stage, anatomical location and patient-related factors, and include surgery, radiotherapy, chemotherapy, and, more recently, immunotherapy (118). However, accumulating evidence indicates that viral status, particularly HPV positivity in OP SCC, influences treatment response, prognosis, and risk stratification (119). In contrast, HPV-negative tumors and most OC SCCs continue to exhibit a more aggressive clinical course and remain strongly associated with traditional risk factors such as tobacco and alcohol exposure (120). Consequently, accurate viral characterization is becoming increasingly relevant for patient stratification and therapeutic decision-making.

Surgery remains the cornerstone of treatment for most patients with resectable OC SCC and selected OP SCC cases. In OC SCC, surgical resection with adequate margins is generally considered the primary treatment modality and is frequently combined with neck dissection depending on nodal involvement and pathological risk factors (118). Although HPV status does not currently alter surgical indications, HPV-positive tumors often present with advanced nodal disease despite smaller primary lesions, which may influence treatment planning and postoperative management (121).

Radiotherapy and concurrent chemoradiotherapy play a central role in the management of locally advanced OP SCC and high-risk OC SCC. Multiple studies have demonstrated that HPV-positive OP SCC exhibits enhanced radiosensitivity and improved locoregional control compared with HPV-negative disease (121, 122). These observations have established HPV status as an important prognostic and predictive biomarker and have contributed to the development of risk-adapted treatment strategies.

The increasing understanding of virus-associated tumor biology has also stimulated interest in immunotherapeutic strategies. Immune checkpoint inhibitors targeting the PD-1 pathway, including pembrolizumab and nivolumab, have demonstrated clinical benefit in recurrent or metastatic HNSCC and are now incorporated into current treatment guidelines (78). HPV-positive tumors frequently display increased immune cell infiltration, enhanced antigen presentation, and elevated expression of immune checkpoint molecules, suggesting a potentially favorable immunological context for immunotherapy (123). In parallel, therapeutic vaccines and antigen-specific approaches targeting HPV-derived proteins, including E6, E7, E1, and E2, are being actively investigated as strategies to enhance antitumor immune responses (124).

Future advances are likely to arise from the integration of viral biomarkers, molecular profiling, and immunological signatures into personalized therapeutic approaches. Standardized viral characterization may further refine patient selection, improve risk stratification, and facilitate the development of precision medicine strategies for virus-associated head and neck cancers.

### 3.10 Limitations of available evidence

The available evidence is limited by substantial methodological heterogeneity across studies, particularly regarding viral detection techniques, diagnostic thresholds, and reporting standards. The use of PCR-based assays, IHC, and ISH limits direct comparability among studies and complicates causal interpretation, especially for non-HPV oncogenic viruses.

Most included studies were observational and retrospective in design, increasing susceptibility to selection bias, information bias, and confounding by established risk factors such as tobacco and alcohol exposure. In addition, the geographic distribution of studies was uneven, with most evidence originating from North America, Europe, and East Asia, whereas low-income regions remained underrepresented. Consequently, the generalizability of global estimates may be limited.

Another important limitation was the relatively low adoption of multimodal viral detection strategies. Although combined molecular and IHC approaches are generally considered more robust for identifying biologically relevant viral infection, only 12% of studies employed multimodal methods. This limitation reduces the ability to reliably distinguish transcriptionally active infection from incidental viral presence, particularly in OC SCC.

Finally, evidence for non-HPV viruses was frequently derived from small cohorts, heterogeneous study designs, inconsistent anatomical classification, and variable outcome reporting. These limitations preclude robust etiological, prognostic or predictive conclusions regarding EBV, HSV, MCPyV, CMV, and other less frequently investigated virus.

## 4 Conclusions

This systematic review demonstrates that HPV is the central and best-supported oncogenic virus in OP SCC, with consistent epidemiological, clinicopathological, molecular, and prognostic evidence across diverse populations. In contrast, the role of HPV in OC SCC, as well as that of other oncogenic viruses in both OC and OP SCC, remains limited, heterogeneous, and largely unsupported by high-quality causal evidence. By synthesizing data from 366 studies, this review provides a site-specific comparative framework for evaluating viral oncogenesis in OC SCC and OP SCC while systematically assessing the contribution of multiple oncogenic viruses beyond HPV. This approach highlights the substantial impact of methodological heterogeneity in viral detection and reporting, and offers a structured basis for interpreting inconsistencies across epidemiological, clinicopathological, and prognostic findings.

Collectively, these findings underscore the need of site-specific interpretation of viral oncogenesis and caution against extrapolating OP SCC-based paradigms to OC malignancies. They further emphasize the importance of standardized diagnostic methodologies, regionally tailored public health strategies, and targeted future research to clarify the role of non-HPV oncogenic viruses in OC and OP carcinogenesis. Improved viral characterization and methodological harmonization will be essential for advancing prevention, risk stratification, and precision management of virus-associated head and neck cancers.

## 6 Future directions

Future research should prioritize the standardizing of viral detection, classification, and reporting strategies, ideally through integrated molecular and IHC approaches capable of distinguishing transcriptionally active infection from incidental viral presence. Large, prospective multicenter studies with precise anatomical site classification and comprehensive adjustment for behavioral, environmental, and host-related risk factors are needed, particularly to clarify the role of non-HPV oncogenic viruses in OC and OP SCC. Expanded investigation in underrepresented geographic regions will be essential to define virus-attributable risks across diverse populations and improve the global applicability of current evidence. Furthermore, the integration of viral genomics, host immune profiling, transcriptomics, and molecular tumor characterization may provide deeper insights into virus-host interactions and the biological mechanisms underlying viral carcinogenesis. Future efforts should also focus on identifying clinically relevant biomarkers that improve viral attribution, prognostic stratification, and therapeutic decision-making. Collectively, these advances may support the development of more effective surveillance, prevention, and precision medicine strategies for virus-associated head and neck cancers.
